# Effectiveness of Multifaceted Lifestyle Interventions in Prediabetic Adults: A Field Study in an Urban Slum Population of Pune, India

**DOI:** 10.7759/cureus.68635

**Published:** 2024-09-04

**Authors:** Kajal Srivastava, Hetal Rathod, Chaitali Borgaonkar, Manisha Rathi, Akhil R, Saurabh Sujanyal

**Affiliations:** 1 Community Medicine, Dr. D. Y. Patil Medical College, Hospital & Research Centre, Dr. D. Y. Patil Vidyapeeth (Deemed to be University), Pune, IND; 2 Physiotherapy, Dr. D. Y. Patil College of Physiotherapy, Dr. D. Y. Patil Vidyapeeth (Deemed to be University), Pune, IND

**Keywords:** yoga, exercise, type 2 diabetes mellitus, early diagnosis, diabetes complications, glycated hemoglobin

## Abstract

Introduction

Adults with diabetes have an increased risk of hypertension, heart attack, and stroke than those without diabetes. Diagnosing prediabetes at an early stage can significantly reduce the risk of diabetes through simple interventions such as lifestyle modifications. Lifestyle modifications such as weight loss combined with regular physical exercise and a healthy diet can help delay or prevent the progression of diabetes. This study aims to estimate the prevalence of prediabetes among the urban slum population and to assess the effect of lifestyle modifications on blood sugar levels, glycated hemoglobin (HbA1c), and lipid profile among the participants.

Methods

A quasi-experimental field study was conducted among the urban slum population. Participants were randomly selected from previous health screening data. Pre-intervention blood evaluations were performed, and those who fulfilled the criteria were enrolled for interventions. The follow-up period lasted three months and included telephonic and in-person meetings for support and motivation. All variables were reevaluated at the end of the follow-up period.

Results

Out of 34 participants included in the study, 20 completed the three-month follow-up. Statistically significant changes were observed after three months of intervention in weight, fasting blood sugar, HbA1c, BMI, triglycerides, and high-density lipoprotein (HDL) cholesterol levels. However, decreases in systolic blood pressure (BP), diastolic BP, total cholesterol, and low-density lipoprotein (LDL) cholesterol were not statistically significant.

Conclusion

The study revealed that lifestyle intervention programs promoting healthy diets, physical activity, and body weight reduction can prevent or delay the onset of diabetes among high-risk populations. The effectiveness of interventions across community settings depends on delivery formats, implementers, and the level of motivation of participants

## Introduction

Diabetes is a chronic disease caused by insufficient insulin production or ineffective use of insulin by the body [1]. Adults with diabetes have an increased risk of hypertension, heart attack, and stroke than people without diabetes. Simple lifestyle changes such as maintaining body weight, physical activity, a healthy diet, and avoiding tobacco and alcohol use can prevent the onset of diabetes [2]. Prediabetes (intermediate hyperglycemia) is a high-risk state for diabetes defined by glycemic variables that are higher than normal but lower than diabetes thresholds. Early diagnosis of diabetes mellitus or prediabetes can be achieved by simple testing of blood sugar levels.

Diabetes is becoming an important public health challenge in India, with 77 million individuals with diabetes in India in 2019, which is expected to rise to over 134 million by 2045. Approximately 57% of these individuals remain undiagnosed [3]. According to the latest National Family Health Survey (NFHS) - 5 data, the prevalence of diabetes in India among males is 15.6%, while in females, it is 13.5% [4]. Between 2000 and 2019, there was a 3% increase in age-standardized mortality rates from diabetes. In lower-middle-income countries, the mortality rate due to diabetes increased by 13% [5].

The World Health Organization (WHO) has defined prediabetes as a state of intermediate hyperglycemia using impaired fasting glucose as fasting plasma glucose of 110-125 mg/dl and glycated hemoglobin (HbA1c) of 5.7-6.4% [6]. According to the Indian Council of Medical Research-India Diabetes (ICMR-INDIAB) study in 2023, which was a cross-sectional population-based survey covering a representative sample of all states and union territories in India, the overall weighted prevalence of diabetes and prediabetes in India was 11.4% and 15.3%, respectively [7]. The weighted prevalence of diabetes (both known and newly diagnosed) in Maharashtra (2016) was 8.4%, with 10.9% in urban areas and 6.5% in rural areas. The prevalence of prediabetes was 12.8%; 15.2% in urban areas and 11.1% in rural areas [7-8].

Most people do not know that they are prediabetic, as there are no clear symptoms. Prediabetes means, in simple terms, that blood sugar levels are higher than they should be but not high enough to be called diabetes. Diabetes has more severe implications than prediabetes and can damage various organs like the eyes, heart, kidneys, nerves, and limbs. Even small changes can have a huge impact on managing this disease or preventing it altogether. Early diagnosis and treatment can help to prevent these complications. Prediabetes can be expanded as “Prevent Diabetes." If we can diagnose prediabetes at an early stage, simple interventions such as lifestyle modifications can reduce the risk of diabetes.

It is already established that lifestyle modifications like weight loss with regular physical exercise and a healthy diet help delay or prevent the progression of diabetes [9]. As most diabetes complications cause symptoms only at a very late stage, these complications can be evaluated during the prediabetes stage. By screening high-risk groups for prediabetes and encouraging them to have a healthy diet and regular exercise of at least 30 minutes a day for most days of the week, the progression to type 2 diabetes mellitus can be prevented. This opportunity can also be used for screening other diseases like hypertension and dyslipidemia, which are risk factors themselves. Therefore, this study was conducted to understand the hidden burden of disease in the community and to assess the role of holistic intervention in improving glycemic parameters and reducing risk factors in an Indian urban slum population.

The objectives of this study were to estimate the proportion of prediabetes among the urban slum population and to assess the effect of lifestyle modification on blood sugar levels, HbA1c, and lipid profile among the population.

## Materials and methods

This quasi-experimental field study was conducted in an urban field practice area of a tertiary care teaching hospital, Dr. D. Y. Patil Medical College, Hospital & Research Centre, Pune, Maharashtra, India. The study was approved by the Ethics Committee of Dr. D.Y.Patil Vidyapeeth (approval number: DYPV/EC/527/2020).

Residents in the age group of 20-60 years with a history of hypertension, a family history of DM type 2, and a history of gestational DM were included in the study. Participants who were known diabetics, pregnant females, severely ill and bedridden, persons with disabilities, on oral hypoglycemic agents, and who did not give consent for intervention were excluded from the study. Participants who were initially enrolled but missed more than two consecutive in-person meetings at the field practice area under the Urban Health Training Center (UHTC) or did not meet the follow-up criteria were excluded

Sample size

Assuming a medium effect size of 0.5, with an alpha error of 0.05 and power of 80 %, the minimum sample calculated was 34. However, we enrolled 40 participants in this study. The software used was G*power 3.1.9.7 [10].

Study participants were selected from the existing health screening data available in the field practice area under the UHTC. A sample size of 40 participants was targeted using a computer-generated simple random sampling table.

Data collection

After obtaining informed written consent from the participants, sociodemographic details and risk factors such as a history of hypertension, family history of DM, history of gestational DM, addictions, physical activity (duration and type), and diet were documented using a pre-tested, pre-validated questionnaire. Further, participants were subjected to physical examination (pulse, blood pressure (BP), pallor, height, weight, BMI, waist circumference, and hip circumference).

BP was measured with Dr. Morepan BP-02 monitor (Morepen Laboratories Limited, Baddi, Himachal Pradesh, India) and a standard calibrated and pre-checked digital weighing machine was used for weight measurement. To prevent variations, the physical examination was conducted by a single evaluator. To ensure the integrity of collected data, we implemented a rigorous quality assurance protocol. Intensive training was conducted for field researchers on standardized measurement techniques and the proper use of calibrated instruments. Further, an observer was employed for critical measurements to minimize bias and error, while also instituting systematic quality control measures including regular supervisory spot checks and periodic instrument recalibration. After explaining the procedure, blood was drawn for estimation of fasting blood sugar (FBS) level and HbA1c; high-performance liquid chromatography (HPLC) method using D-10 Hemoglobin Testing System (Bio-Rad Laboratories, Inc., Hercules, California, United States) was used for HbA1c estimation. The lipid profile was evaluated using the Dimension EXL 200 (Siemens AG, Munich, Germany). Those participants who were found to have blood sugar levels between 110 mg/dl and 125 mg/dl, HbA1c values between 5.7% and 6.4% [1], history of hypertension, strong family history of DM, and BMI more than 23 [11], were enrolled for intervention. Participants with blood sugar levels of more than 125 mg/dl and HbA1c of 6.5% or more were referred for further evaluation and management as they met the diagnostic criteria for diabetes.

Study process

A total of 40 participants were recruited using a computer-generated simple random sampling table, based on existing screening data at the Urban Health Training Centre (UHTC). Of the 40 participants targetted, three participants did not give consent for baseline blood parameter evaluation and three did not show up for the initial evaluation. This reduced the number of participants to 34, all of whom had baseline data collected and were enrolled in the study.

Study participants were given training on interventions (yoga, aerobic exercise, dietary modifications) by experts in the field. Follow-up evaluation was done after three months. Telephonic follow-ups were done thrice weekly by investigators to motivate participants along with once-weekly in-person meetings. Required advice and psychological support were given by investigators during telephonic interviews as well as during in-person meetings. A total of 14 participants missed more than two consecutive in-person meetings at UHTC or did not meet the follow-up criteria and were excluded. After three months of intervention, 20 participants completed the study and were included in the final analysis.

**Figure 1:**
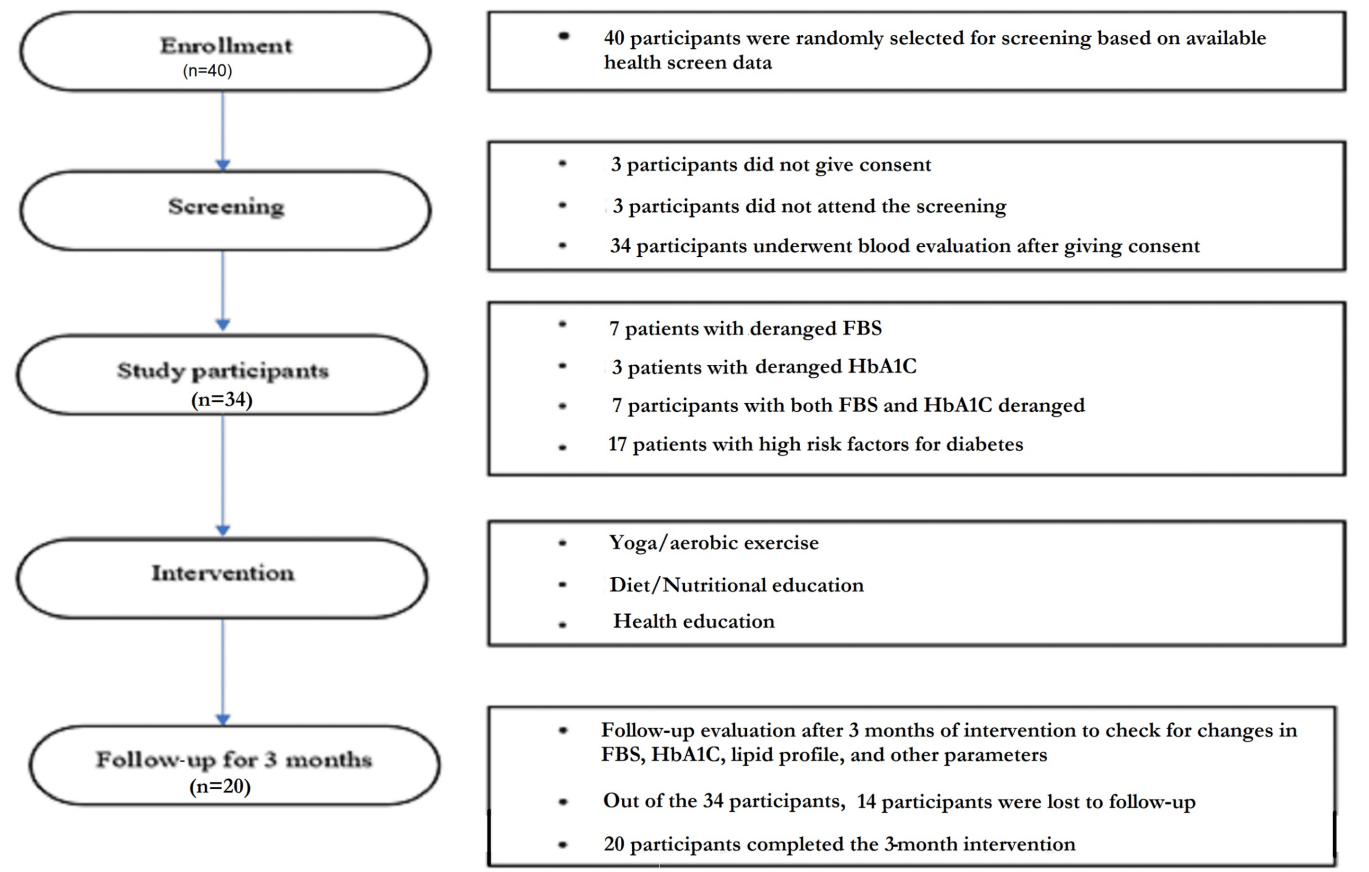
Design outline and data flowchart FBS: fasting blood sugar; HbA1c: glycated hemoglobin

All study variables were reevaluated at the end of the intervention period (Figure 1). There were no adverse events reported among the participants during the study period. 

Operational definitions

FBS: Capillary blood was taken after eight hours of fasting. American Heart Association (AHA) guidelines (2019) were used for diagnosis. An FBS level of 100-125 mg/dl was considered prediabetes; more than 126 mg/dl is considered diabetes and less than 100 mg/dl is considered normal [1].

HbA1c: The HbA1c test measures the average blood sugar for the past two to three months [1]. The normal value was considered less than 5.7%. If the value was 5.7-6.4%, it was considered to be prediabetes stage, and diabetes was diagnosed if HbA1c was 6.5% or higher.

Lipid profile: Total cholesterol, triglycerides, HDL cholesterol, very LDL (VLDL) cholesterol, and LDL cholesterol were evaluated. For total cholesterol estimation, values were considered as follows: normal if the value was less than 200 mg/dl, borderline high if the value was 200-239 mg/dl, and high if the value was 240 mg/dl and above. For triglycerides, the normal value was taken as less than 150 mg/dl, borderline high if the value was 150-199 mg/dl, high if the value was 200-499 mg/dl, and very high if the value was more than 500 mg/dl. HDL values of more than 40 mg/dl in males and more than 50 mg/dl in females were considered normal. VLDL value less than 30 mg/dl was considered normal in both males and females. For LDL cholesterol levels, less than 100 mg/dl was considered optimal, near optimal/above optimal if the value was 100-129 mg/dl, borderline high if the value was 130-159 mg/dl, high if the value was 160-189 mg/dl, and very high if the value was 190 mg/dl or more [12].

The blood sugar level and HbA1c status of the study participants were not disclosed to co-investigators who were involved in interventions, telephonic interviews, and in-person meetings.

Data analysis

The data were entered into Microsoft Excel 2022 (Microsoft Corporation, Redmond, Washington, United States) and analyzed using EpiInfo software 2011 (CDC, Atlanta, Georgia, United States), and MedCalc statistical software version 18.2.1 (2018; MedCalc Software Ltd, Ostend, Belgium). Categorical variables were expressed in terms of frequency and percentages (where applicable), and continuous variables were expressed as mean±SD and Median (interquartile range (IQR), where applicable. Normal distribution was verified by the Shapiro-Francia test. A paired t-test/Wilcoxon test was used to check for the significance of pre and post-intervention observations. In all the tests performed, P < 0.05 was considered to be statistically significant.

## Results

In this study, 40 participants were selected for the screening. At the beginning of the field trial, 34 participants were included but only 20 participants completed the three months follow-up. Participants who missed more than two consecutive in-person meetings at UHTC were excluded from the study.

In this study population, the majority of the population, 12 (60%) were females. The mean age of the participants was 40.20 years with a mean height of 156.80 cm. Regarding the type of family, 15 (75%) participants belong to nuclear families (Table 1).

**Table 1:** Socio-demographic details of the study participants. SD: standard deviation; CI: confidence interval; n: frequency

Variables	Mean±SD	95% CI
Age of participants (in years)	40.20±9.21	35.89 - 44.51
Height of participants (in cm)	156.80±6.06)	153.96 - 159.64
Sex (n=20)	n (%)	95% CI
Female	12 (60)	36.05-80.88
Male	8 (40)	19.12-63.95
Type of family (n=20)		
Joint	5 (25)	8.66-49.10%
Nuclear	15 (75)	50.90-91.34

Risk factors like a history of hypertension, family history of DM, and sedentary/very little physical activity were found in one (5%), four (20%), and six (30%) individuals, respectively. Smoking/tobacco consumption and alcohol consumption were found in one (5%) participant in each (Table 2).

**Table 2:** Distribution risk factors for diabetes mellitus among study participants CI: confidence interval; n: frequency

Variables	n (%)	95% CI
History of Hypertension (n=20)		
Yes	1 (5)	0.13-24.87
No	19 (95)	75.13-99.87
Family history of diabetes (n=20)		
Yes	4 (20)	5.73-43.66
No	16 (80)	56.34-94.27
Smoking/Tobacco consumption (n=20)		
Yes	1 (5)	0.13-24.87
No	19 (95)	75.13-99.87
Alcohol consumption (n=20)		
Yes	1 (5)	0.13-24.87
No	19 (95)	75.13-99.87
Physical activity (n=20)		
Heavy physical activity	1 (5)	0.13-24.87
Moderate physical activity	13 (65)	40.78-84.61
Sedentary/ Very little physical activity	6 (30)	11.89-54.28

Changes in the parameters are listed in Table 3, which compares the effect of three months of intervention on the study population. There was a statistically significant change after three months of intervention in weight, FBS, HbA1c, BMI, triglycerides, and HDL cholesterol levels whereas there was no statistically significant change found in systolic BP, diastolic BP, total cholesterol, and LDL cholesterol after three months of intervention (Table 3).

**Table 3:** Pre- and post-intervention evaluation of parameters among the study participants * Paired t-test was performed (Mean±SD was used for analysis) ** Wilcoxon signed rank test was performed (Median (IQR) was used for analysis) *** P<0.05 is considered as statistically significant BMI: body mass index; FBS: fasting blood sugar; HbA1c: glycated hemoglobin; BP: blood pressure; HDL: high-density lipoprotein; LDL: low-density lipoprotein; SD: standard deviation; df: degree of freedom

Variables	Pre-intervention, (n=20)	Post-intervention (n=20)	Significance		
			t	df	P- value***
Weight*	73.60±13.21	71.95±12.81	-3.208	19	0.005
BMI**	28.95 (26.82- 31.44)	28.36 (26.02- 30.78)	-	-	0.011
FBS*	101.55 (10.84)	93.70 (10.16)	-4.958	19	< 0.001
HbA1c**	5.80 (5.30 - 6.15)	5.20 (5.0 - 5.80)	-	-	< 0.001
Systolic BP*	120.40 (13.79)	122.40 (11.19)	1.209	19	0.241
Diastolic BP*	75.90 (9.28)	74.20 (5.02)	-0.917	19	0.371
Triglycerides**	110 (81 - 143)	98 (81 - 141)	-	-	0.049
HDL cholesterol**	37.50 (31.50 - 46)	37 (33.50 - 48.50)	-	-	0.024
Total cholesterol**	172.50 (156- 190.50)	170 (159 - 180)	-	-	0.623
LDL cholesterol**	107 (96.50 - 124)	109 (93.50 - 126.50)	-	-	0.621

## Discussion

Prediabetes is a state of abnormal glucose homeostasis in which blood glucose levels are elevated above those considered normal, but not high enough to meet the criteria required for a diagnosis of diabetes and is characterized by IFG and/or impaired glucose tolerance (IGT) [13]. Macrovascular complications including cerebrovascular disease, coronary artery disease, peripheral artery disease, and microvascular complications such as diabetic retinopathy, diabetic nephropathy, and diabetic neuropathy are the greatest contributors to diabetes-related healthcare expenditures. Prediabetes increases the risk of these complications, thereby contributing substantially to healthcare expenditures [14]. Hence, to curtail these complications and costs, it is important to diagnose prediabetes and treat it in the first place. Unfortunately, even though evidence-based treatment options, including pharmaceutical and surgical methods, are readily available, clinicians are not fully utilizing them to address prediabetes. This underutilization likely arises from the perception that prediabetes is merely a risk factor and not a significant medical condition on its own. Similarly, individuals with prediabetes often hesitate to accept antidiabetic prescriptions due to not having diabetes, which further contributes to clinician reluctance in initiating drug treatment for prediabetes. Lifestyle modifications including exercise and nutritional education can play a significant role in the treatment of prediabetes as they are not only efficient but also quite acceptable to the patients.

The present study consisted of 20 participants and pre-intervention, the mean FBS level was 101.55 mg/dl. After three months of intervention, the mean FBS was found to be 93.70 mg/dl. This change was statistically significant with t = -4.958 and p = 0.0001. This was consistent with Lindstrom et al.'s study which showed a significant difference in FBS levels (−0.2 mmol/L) in the intervention group [15]. Also, a prospective randomized controlled trial (RCT) was studied by Hu et al. in 2017 where the study primarily focused on prediabetic Chinese adults who lived in rural China. Hu et al. concluded a statistically significant change in FBS of -3.9 mg/dl in the intervention group (p < 0.001) [16].

Similarly, a longitudinal study consisting of 66 individuals was carried out by Defeudis et al. evaluating the efficacy of using diabetes conversation maps with a weight loss program [17]. The study was carried out for three months and concluded a significant reduction in HbA1c (p < 0.0001) for the intervention group compared to the control. Additionally, Yang et al.'s RCT (n = 60) demonstrated a significant reduction in HbA1c at a 12-month follow-up (p < 0.05) in the intervention group compared to the control [18]. This is consistent with the finding in our study where in the pre-intervention results, the mean HbA1c was 5.80. When the participants were evaluated after three months of intervention including exercise and nutritional education, the mean HbA1c was found to be 5.20 and was statistically significant (p-value <0.0001).

Currently, the primary approach to managing coronary artery disease involves medication. Nevertheless, drug therapy has its constraints. Physical activity has a dual impact: it benefits individuals with dyslipidemia and enhances their lipid profiles. Numerous research trials have established a relationship between aerobic exercise and levels of HDL cholesterol, LDL cholesterol, and triglycerides. A recent RCT was conducted by Nybo et al. with 36 male participants for three months and concluded a significant increase in HDL cholesterol (0.1 mmol/L) and a decrease in LDL cholesterol (0.1 mmol/L) [19]. Similarly, Kraus et al.'s recent RCT which included 111 participants (both men and women) for 24 weeks, showed a significant increase in HDL cholesterol (4.3 mg/dl), a decrease in LDL cholesterol (1.9 mg/dl), and a decrease in triglycerides (28.4 mg/dl) [20]. On the other hand, our study reported a 0.5 mg/dl decrease in HDL cholesterol (p = 0.0237), a 2 mg/dl increase in LDL cholesterol (p = 0.6215), and a 12 mg/dl decrease in triglycerides (p = 0.0494). These differences in the results can be attributed to variations in study design, participant weight, and study population as a whole.

Our study also reported a significant post-interventional decrease in average weight and BMI of about 1.65 kg (p = 0.0046) and 0.59 kg/m^2^ (p = 0.0110) respectively. These results align with Hoogendoorn et al.'s pre-post interventional study which involved 56 highly motivated Dutch adults who had type 2 DM (n = 30) or were at risk for type 2 DM (n = 26). It reported a significant decrease in BMI of 1.1 kg/m^2^ (p < 0.01) [21]. Similarly, a smaller non-RCT conducted in Australia, which included 26 participants, investigated the impact of an educational intervention and a highly restricted calorie diet. This study reported a substantial and statistically significant average weight loss of 6.6 kg (p = 0.004) [22].

A meta-analysis of 10 RCTs that included data on 1880 individuals with prediabetes demonstrated that individuals who received lifestyle intervention for one year had a 54% lower risk of progressing to type 2 diabetes than people receiving treatment as usual (4% vs. 10%; relative risk (RR) 0.46 (95% CI 0.32, 0.66)) [23]. After three years of follow-up, pooled results of 11 RCTs involving a total of 5224 people with prediabetes demonstrated that lifestyle intervention participants had a 36% lower risk of developing type 2 diabetes compared with those receiving treatment as usual (14% vs. 23%; RR 0.64 (95% CI 0.53, 0.77)) [23]. This shows that prediabetes is a major but silent incubator of future morbidity. Therefore, both clinicians and patients need to raise their voices to acknowledge the escalating burden of this condition. The primary approach to managing prediabetes should focus on lifestyle modification, while pharmacological therapy can be employed if necessary to break the harmful cycle of pathophysiological processes that can lead to complications and the development of diabetes. More research is needed to thoroughly understand the connection between the duration of lifestyle interventions and their effectiveness in influencing indicators related to prediabetes and/or type 2 diabetes in specific age groups.

There are some limitations of this study. The study was designed mainly for the urban slum population, but due to the lack of motivation, lack of space for exercise, working hour constraints, and family commitments a substantial proportion of study participants were lost to follow-up. Females were the predominant participants in the trial, and their nature of work and family constraints affected the outcome. As a result, further research with a motivated and higher sample size is required to generalize the study's findings.

## Conclusions

The study revealed that lifestyle intervention programs promoting healthy diets, physical activity, and body weight reductions can prevent or delay the onset of diabetes among high-risk populations. At-risk individuals need education, ongoing support, and an environment conducive to following healthy behaviors. We learned from the study that the effectiveness of interventions across community settings depends on delivery formats, implementers, and the level of motivation of participants. Further field studies in different socioeconomic settings will reveal the influences and strengths of physical activity and lifestyle modifications on blood sugar levels.
